# Prevention and Management of Infectious Complications of Retrograde Intrarenal Surgery

**DOI:** 10.3389/fsurg.2021.718583

**Published:** 2021-08-09

**Authors:** Johnathan A. Khusid, John C. Hordines, Areeba S. Sadiq, William M. Atallah, Mantu Gupta

**Affiliations:** ^1^Department of Urology, Icahn School of Medicine at Mount Sinai, New York, NY, United States; ^2^Department of Urology, SUNY Downstate College of Medicine, Brooklyn, NY, United States

**Keywords:** infection, ureteroscopy, nephrolithiasis, urology, sepsis

## Abstract

Kidney stone disease (KSD) is a commonly encountered ailment in urologic practice. Urinary tract infection (UTI) is commonly associated with KSD, both as an etiology (e.g., struvite and carbonate apatite stones), and as a complication (i.e., obstructive pyelonephritis and post-operative UTI). Indeed, a significant portion of the economic burden of KSD is skewed toward stones associated with infection. UTI is the most common post-operative complication related to stone intervention with progression to urosepsis as a rare but serious consequence. Risk for infection is influenced by a variety of factors including co-morbid conditions, anatomic abnormalities, prior surgical procedures, and local anti-microbial susceptibility. Understanding these risks and the proper steps to mitigate them is an essential component in reducing post-operative morbidity and mortality. Retrograde intrarenal surgery is routinely used for the treatment of KSD. The objective of this review article is to examine the current literature and guidelines for the prevention and management of stone-related infectious complications associated with retrograde intrarenal surgery. Special attention will be given to the incidence, etiology, and antibiotic prophylaxis choice in the management of stone-related infections. Intraoperative risk mitigation techniques will be discussed in conjunction with the management of post-operative infections. Antibiotic stewardship and the potential benefits of reduced empiric antibiotic treatment will also be discussed.

## Introduction

Kidney stone disease (KSD) is a commonly encountered ailment in urologic practice, with an estimated incidence and prevalence in the United States of 0.9 and 8.8%, respectively (1, 2). The prevalence of KSD has been trending upwards in recent years in both population-based and large-scale studies (1, 3). While the increasing rates of KSD can be partly attributed to improvements in imaging technology and detection, the increasing obesity rates in the United States are another likely contributing factor (1, 3).

Urinary tract infections (UTI) are commonly associated with KSD as both an etiology and complication. Kidney stones that form secondary to infection with urease producing bacteria are often referred to as infection stones and common causative organisms include *Proteus, Klebsiella*, and *Staphylococcus* species (4). However, one recent study by Parkhomenko et al. evaluated the urine and stone cultures in a 1,191 patient cohort and found the bacteriology of struvite stones had shifted toward non-traditional urea-splitting microorganisms such as *Enterococcus* species (5). Infection stones often consists of magnesium ammonium phosphate (struvite) or carbonate apatite (6). These stones form from the breakdown of urea into ammonia and carbon dioxide (CO_2_) by urease (6). The increased concentration of ammonia (and later ammonium) creates a locally alkaline environment that facilitates stone formation (6). The increased concentration of CO_2_ drives in the conversion of CO_2_ to carbonate which in turn results in the formation of carbonate apatite (6). Notably, infection stones can be polymicrobial with the incorporation of the non-urease producing bacteria as well (4). Infection stones are more likely to occur in patients with indwelling catheters, neurogenic bladders, or other medical comorbidities that may result in urinary tract microbial colonization (6).

Distinct from *infection stones (IN stones)* are *infected stones (ID stones)* (6). ID stones are colonized kidney stones in which stone genesis and growth is not driven by urease production (6). For example, a kidney stone may form by other metabolic processes (i.e., hypercalciuria) and subsequently become colonized by urinary tract bacteria (6). Another proposed mechanism for the genesis of ID stones is that urinary tract bacteria themselves serve as a stone nidus and host metabolic abnormalities subsequently drive stone growth (6). ID stones are more likely than IN stones to exhibit discordance between stone cultures and urine cultures (6). Contrarily, given that IN stones are often a sequalae of a preceding UTI, stone cultures and urine cultures are often concordant (6). Importantly, IN stones pose a clinical challenge because antibiotics are unable to penetrate the matrix of the stone, making complete surgical extraction crucial (6). If possible, stone fragments should be collected under sterile conditions to be sent for stone culture (7). Stone cultures are not only a better predictor of serious postoperative infectious complications, but they can also provide essential information to guide antimicrobial treatment if a patient develops sepsis (7).

The management of KSD is multimodal, with retrograde intrarenal surgery (RIRS) as a mainstay surgical management option (8). According to current American Urologic Association (AUA) guidelines, patients with a stone burden of <20 mm (or <10 mm for lower pole stones) can be offered RIRS as a first line surgical treatment with excellent stone free rates (9). Other options for surgical management of nephrolithiasis include shock wave lithotripsy (SWL) and percutaneous nephrolithotomy (PCNL) (8). However due to improvements in ureteroscope technology such reduction in scope size and increased laser efficacy, RIRS has become the most commonly utilized surgical management tool for KSD (8, 10).

Though generally safe, important, and potentially morbid complications of RIRS include urinary tract infections and urosepsis. Infectious complications can occur when treating all types of kidney stones including infected, infection, and metabolic stones. Several large collaborative groups have studied infectious complications associated with RIRS. The Clinical Research Office of the Endourology Society (CROES) evaluated 11,885 patients undergoing ureteroscopy and found that postoperative fever occurred in 1.8% of patients with 1.0% of patients developing a UTI and 0.3% of patients becoming septic (10). The Reducing Operative Complications from Kidney Stones (ROCKS) collaborative reported that in 1,817 ureteroscopy procedures, 2.4% of patients were hospitalized secondary to an infectious complication (11). In addition to potential patient morbidity, postoperative sepsis represents a large financial burden on the healthcare system. Although cost calculations can vary widely, Arefian et al. reported that the management of a septic patient incurs a mean total hospital costs upwards of $30,000 per patient (12). Minimizing postoperative infectious complications after RIRS is an important potential avenue for much needed cost-saving as the overall estimated economic impact of nephrolithiasis was 4.5 billion dollars in the employed population of the United States in the year 2000 (13). Given the medical and economic burden produced by infectious complications of RIRS, the aim of this Review is to summarize the literature on infectious complications of RIRS and provide up-to-date clinical mitigation strategies involving pre-operative, intra-operative, and post-operative care.

## Pre-Operative Considerations

The preoperative workup for a patient undergoing RIRS for KSD should include a thorough history and physical exam, basic preoperative bloodwork including basic metabolic panel (BMP) and a complete blood count (CBC), and in most patients, preoperative evaluation by a general medical doctor. Additionally, the AUA guidelines recommend obtaining a urinalysis in all patients, and urine culture in patients in whom there is clinical or laboratory signs of infection (14). The European Association of Urology (EAU) recommends that a preoperative urine culture be obtained for all patients undergoing a procedure for stone removal (15). While positive nitrites on a urinalysis are specific for the presence of bacteria, many uropathogens do not produce nitrates, such as *Enterococcus* (14, 16). Furthermore, many patients with nephrolithiasis have sterile pyuria due to local inflammation and trauma from the stone. Given these pragmatic challenges with using urinalysis alone, in clinical practice, obtaining a urine culture prior to endourologic intervention in all patients is non-controversial (17).

Preoperative urine cultures are an important predictor of infectious complications following RIRS (18–20). Blackmur et al. reported a significant relationship between pre-operative positive mid-stream specimen urine (MSSU) and the incidence of urosepsis, even despite antibiotic prophylaxis (21). A recent meta-analysis conducted by Sun et al. that included 14 studies with 9,532 total patients evaluating potential risk factors for infectious complications following ureteroscopy reported positive pre-operative urine culture to be the most significant predisposing factor for infectious complications (19). **The compilation of evidence makes the use of routine pre-operative urine cultures in all patients strongly encouraged** (7, 22). Routine preoperative urine culture in all patients already appears to be widespread and its value in outcomes prediction for infectious complications support its continued practice.

Urine culture results can fall into one of three general categories: negative, positive, and contaminated. For patients with negative urine cultures, preoperative antibiotic prophylaxis beyond the standard perioperative antibiotic dosing on the day of surgery is generally not indicated. Indeed, the current AUA best practice statement states, “There is no high-level evidence to support the use of multiple doses of antimicrobials in the absence of preoperative symptomatic infection” (14). For patients with positive urine cultures, treatment with culture specific antibiotics should be initiated and follow up urine culture should be obtained. The literature suggests that that RIRS should only be carried out in the presence of a negative follow up culture for patients with a positive preoperative culture (20, 22–24). For patients with persistent positive cultures, practitioners should consider obtaining an infectious disease consultation. For patients with contaminated urine cultures, a repeat sample should be obtained. Contaminated cultures may be reported by the microscopy lab as such, but are also suggested by the presence of epithelial cells on urine microscopy (25). Indeed, the AUA best practice statement recommends that additional samples be obtained from the patient as a midstream sample or *via* catherization for repeat urine studies (14).

*Escherichia coli*, a gram negative rod is, one of the most commonly encountered infectious organisms in the genitourinary system (7, 26, 27). Senocak et al. found in their retrospective review that *E.coli* was not only responsible for the majority of positive overall positive cultures, but also the highest proportion of multi-drug resistant cultures as well (28). Other commonly encountered gram negative organisms include *Proteus mirabilis, Klebsiella* and *Pseudomonas aeruginosa* (4, 7, 26). *E.coli, Proteus* and *Pseudomonas* are known gram negative biofilm formers as well (26). Gram positive organisms tend to consist of *Enterococcus* species and *Staphylococcus aureus* (7, 26) *Enterococcus* species and *Staphylococcus aureus* have also been isolated from biofilms found on catheters of the urinary tract (26). Gram positive bacteria can make up as much as 40% of encountered UTIs in an inpatient setting, with *Enterococcus* making up the majority of these specimens (29). *E.coli* and *Proetus* are of particular interest because they tend to cause infection as a consequence of overgrowth of endogenous flora rather than as foreign invaders (4). Proteus is typically found as part of the gut flora with occasional cross-over to the urethra, but it usually does not cause UTIs in patients with unobstructed urinary tracts (4). The presence of an indwelling catheter allows for ascension of the organisms into the upper urinary tract via unique “swarming” motility (4). Proteus also is a model urease producing organism and is commonly associated with struvite and staghorn calculi (4).

The rise of MDR bacteria is a cause for significant concern and has the potential to increase morbidity and mortality of RIRS. Senocak et al. reported a prevalence of 32.3% for MDR bacteria in pre-operative urine cultures for patients undergoing RIRS for KSD (28). Additionally they found on multi-variate analysis the presence of MDR organisms to be a strong predictor for infectious complications, with an odds ratio of 4.75 after controlling for other patient factors (28). This is despite the use of appropriate preoperative antibiotic therapy (28). Patel et al. reported similar results for PCNL (30). This highlights the importance of antibiotic stewardship and limited use of empiric therapy and preferred use of direct, targeted definitive therapy in the face of a known infection.

Another crucial entity for the urologic practitioner to be aware of prior to RIRS is funguria. Funguria most often is due to *Candida* species and is known as candiduria (31). Although other fungal species such as *Cryptococcus* or *Aspergillus* can infect the kidney, they typically only do so when part of a disseminated infection and rarely cause isolated urinary tract symptoms (31). Funguria often presents as sterile pyuria on urinalysis. Urine cultures are routinely used to diagnose fungal infections with similar efficacy as bacterial infection (31). Like bacterial infections, susceptibility to antifungal agents should be determined and treatment tailored if those services are available. Other routine laboratory tests are less useful in the management of a fungal infection (31). Patients with asymptomatic candiduria are typically not treated unless the patient is scheduled to undergo a urologic procedure (31). Patients with candiduria should be treated with oral fluconazole or IV amphotericin B for several days prior to and following RIRS (14). A longer course of antifungal treatment is recommended in neutropenic patients who present with an obstructive uropathy and are undergoing genitourinary tract surgery such as RIRS (14). Additionally, diabetic patients are more likely to present with candiduria, so a higher degree of clinical susception should be used when treating diabetics (31). Furthermore, a detailed history of recent antibiotic use should be obtained as the loss of saprophytic flora with prolonged use of common antibiotics such as fluroquinolones, third generation cephalosporins, and clindamycin is associated with an increased risk of fungal infections (32). While rare, fungal infections following RIRS have been reported in patients on prolonged antibiotic therapy (33).

In urologic practice, indwelling urinary tract drains are commonly utilized. These include bladder catheters, ureteral stents, and percutaneous nephrostomy tubes. The presence of preexisting drains at the time of RIRS has been found to be associated with post-operative infectious complications (7, 34). The presence of a foreign body in the genitourinary system essentially provides a scaffold for microorganisms to colonize and form a biofilm, acting as nidus for infection (22). In short, a biofilm is a matrix of extracellular material excreted by microorganisms, typically bacterium, that form a film or coat on the surface of a foreign body and allows for the adhesion and further colonization (22, 35). This is particularly relevant for the urologist as many common uropathogens are adept at biofilm formation (26). The manipulation of a foreign body with biofilm during RIRS could seed bacteria throughout the genitourinary track (22).

Indwelling bladder catheters (aka Foley catheters) are commonly encountered in urologic practice and often lead to nosocomial infections called catheter-associated urinary tract infections (CAUTI) (26). A CAUTI is the most commonly encountered hospital-acquired infection in clinical practice (26). Unsurprisingly, indwelling bladder catheters have been shown to be associated with increased risk for infection following RIRS (36, 37). Additionally, indwelling bladder catheters have also found to be strongly associated with pre-operative funguria and the development of SIRS following RIRS (38). Urinary catheters are quickly colonized by bacteria after insertion, and ascent to the bladder takes only 1–3 days (39). The duration of catherization is greatest risk factor for infection (39). Almost all patients with an indwelling catheter for longer than 1 month will have bacteriuria (40). Keeping this in mind, catheters would ideally be changed as close to the procedure as possible. Patients with indwelling catheters with asymptomatic bacteriuria should be treated prior to the procedure (39). Furthermore, obtaining a urine culture from a “freshly” exchanged catheter may help better tailor antimicrobial prophylaxis during RIRS. Biofilms formed on catheters tend to be polymicrobial if they have been in place for more than a few days (40). A “freshly” exchanged sample could avoid contamination and may give more relevant clinical data.

Several studies have established an association between the presence of pre-operative stents and infection following RIRS (19, 34, 36). The recent meta-analysis by Sun et al. found that pre-operative ureteral stents are significantly associated with the development of infectious complications following RIRS with an odds ratio of 1.53 (19). Like indwelling bladder catheters, ureteral stents are rapidly colonized and subject to biofilm formation shortly after they are placed (23). Importantly, stent related infection can occur in the absence of biofilm formation, indicating that other mechanisms also mediate the relationship between pre-operative ureteral stents and the development of sepsis after RIRS (22). Urine cultures are often discordant with stent cultures, making antibiotic selection challenging (23). Nevo et al. found 11% of patients had positive stent cultures despite a sterile urine culture, and 26.4% patients with positive urine and stent cultures had discordant cultures (23). The same study also demonstrated an association between positive stent cultures and post-procedure sepsis (23). Nevo et al. found a significant relationship between prolonged stent dwelling time and postoperative sepsis in patients who underwent ureteroscopy after stent insertion (35). Indeed, Nevo et al. reported a fivefold increase in urosepsis risk for patients with indwelling stent times longer than 30 days as compared to patients with indwelling stent times shorter than 30 days (35). An increase in sepsis rates were also observed at 2, 3 and >3 months of stent dwell time (35). Though these findings suggest that stent exchange prior to RIRS should be considered for patients with longer indwelling stent times, there are currently no prospective randomized controlled trials on which to base definitive recommendations. These studies suggest that in patients with indwelling stents, a higher degree of suspicion for infectious complications should be maintained despite sterile preoperative urine cultures and sending stent cultures should be considered intraoperatively. Pragmatically speaking, unlike foley catheters, stents cannot be routinely exchanged prior to RIRS, given that stent exchanges are usually performed in the operating room. “In-office” stenting has be adapted by some urologists and if office-based stenting were to gain widespread adaptation, routine stent exchange prior to RIRS may be a potential avenue for minimizing complications from RIRS in the future (41). Drug-eluting and anti-microbial coated stents have been explored as a means to address infection concerns, but currently there is no widely adapted drug eluting or coated ureteral stent (22, 26). This represents another important avenue for future research.

Patients may undergo percutaneous nephrostomy tube placement prior to RIRS for a variety of reasons. Most commonly, these are acutely ill patients who are too unstable to undergo retrograde ureteral stent placement or patients in whom retrograde renal access could not be established (42). Preexisting PCNs are a known risk for infectious complications following RIRS (36). However, given that patients who undergo PCN placement rather than stenting for acute obstruction are often sicker, it is unclear if the higher sepsis rates at the time of RIRS are related to the actual PCN, or to severity of illness at the time of initial decompression (42). Like ureteral stents, PCNs cannot be pragmatically exchanged prior to RIRS in most practice setting. One systematic review concluded that PCN urine cultures can help guide antibiotic selection when selecting antibiotics for the treatment of sepsis in the context of upper urinary tract obstruction (43). Ideally these cultures should be drawn at the time of decompression, and a general rule, cultures should be taken from the drain and never from the collection bag. This applies to foley catheters as well. Of note, though PCN cultures can help guide antibiotic selection, there is little utility in treating to sterility as there were no differences in infectious complication outcomes for patients who waited to have urine from their PCN sterilized before undergoing upper tract stone surgery (43). For patients who present septic and undergo emergent decompression, either with a stent or PCN, there is no well-established, evidence-based guideline for how much time should elapse prior to undergoing definitive RIRS. However, it is reasonable and intuitive to allow for completion of treatment course of antibiotics for a complex urinary tract infection, which is at least 7 days (44).

The risk factors for developing infectious complications following RIRS have been extensively studied and several at risk populations have been identified. In addition to positive pre-operative urine culture and indwelling urinary tract drains, these risk factors include female gender, diabetes, renal abnormalities, ischemic heart disease, advanced age, history of recurrent UTI, previous incomplete stone removal, urinary diversion, paraplegia, and a higher Charleston comorbidity index (11, 18, 19, 22, 34, 45, 46). Immunosuppression, recent chemotherapy or steroid treatment, poor nutrition and prolonged hospital course are other factors that increase the risk of post-operative infectious complications, in general (47). Some of these populations require special consideration in preparation for RIRS.

Female gender is a well-established risk factor for infectious complications following RIRS (19, 37, 48). The shorter urethra puts the female urinary tract at higher risk for colonization with perineal bacteria and rectal bacteria that can cause infection (19, 39, 47). Clinicians should maintain a higher index of suspicion for infectious and infected stones in female patients. For pregnant patients, clinicians must be wary to avoid potentially teratogenic antibiotics such as fluoroquinolones and aminoglycosides (49). Although ureteroscopy is considered safe, pregnant patients are maintained as a high risk population by the AUA (50, 51). One meta-analysis reported that there were no increased rates of complications following RIRS for pregnant patients, and complications were typically minor when encountered (50). A second retrospective review also noted no difference in complication rates between pregnant and non-pregnant patients (52). Pregnant patients may see a delay in diagnosis of KSD in favor of other medical or obstetric causes (50). Stenting following RIRS in a pregnant patients may be problematic because the higher concentrations of calcium and urate in the urine increase the risk of stent encrustation (53). Stents are also more prone to migrate in pregnant patients (53). If the pregnancy is considered high risk or there are unique obstetric concerns, consider an OB/GYN consult prior to surgery and/or fetal heart monitoring with an OB/GYN present during surgery.

Sun et al. that reported that diabetes mellitus was among the most clinically relevant pre-operative risk factors for infectious complications after undergoing ureteroscopy (19). Li et al. also found diabetes mellitus to be an independent predictor of infection following RIRS (45). Patients with diabetes mellitus are more susceptible to infection for several reasons (19). Higher glucose in the urine may act to facilitate bacterial survival and proliferation within genitourinary system although evidence supporting a direct relationship is lacking (54). Furthermore, impaired immune function secondary to incomplete phagocytosis and diminished function of granulocytes in diabetic patients can leave them more prone to infections (54). Additionally, diabetic patients are prone to developing diabetic cystopathy which may result in recurrent UTIs secondary to incomplete bladder emptying (55). Patients with diabetes mellitus as also at a higher likelihood of developing a UTI compared to the general population outside the context of post-surgical complications (54).

Prior to surgery, known diabetic patients should be evaluated with an HbA1c, as those with higher HbA1c levels could be at higher risk for complications and longer hospital stays (51). One might consider delaying elective RIRS if blood glucose exceeds 400 mg/dL preoperatively (51). Patients should be counseled on how their diabetic medications regime should be altered on the day of surgery (51). Overall, a higher degree of suspicion should be maintained for infectious complications postoperatively for diabetic patients. If blood glucose remains uncontrolled or the patient's medication regime is complex, consider an endocrinology consultation.

Patients artificial joints placed within 2 years of the procedure should be considered for antibiotic prophylaxis for procedures that can cause bacteremia such as RIRS, however they are not considered independently high risk for infectious complications by the AUA (14). Other comorbidities such as advanced age, ischemic heart disease, and higher Charleston comorbidity index have been associated with greater risk of post-operative infectious complications after RIRS (11, 18, 22, 34). For elderly patients, preoperative evaluation by a geriatrician should be considered and for patients with abnormal cardiac histories, preoperative evaluation by a cardiologist should be considered. As a general rule of thumb, a multidisciplinary approach with specialist consultation should be utilized for the preoperative optimization of patients with multiple co-morbidities. Pre-operative recommendations are summarized in Table 1.

**Table 1:** Summary of pre-operative considerations.

**Risk factor**	**Mitigation strategy**
Positive preoperative urine culture (bacteria)	Culture specific antibiotics
Positive preoperative funguria	Oral fluconazole or IV antifungal for several days prior to RIRS (14). Extended therapy in neutropenic patients (14).
Presence of indwelling drain	•**Foley**: Asymptomatic bacteriuria treated prior to RIRS, cultures obtained ideally from a recently exchanged catheter (39). Ensure that the culture is drawn directly from the drain and not the collection bag. •**Ureteral stent**: Limit dwell time to one month if possible, consider exchange if lengthy time to definitive therapy anticipated (35). •**PCN**: Obtain renal pelvis culture to guide antibiotic selection (43). Ensure that the culture is drawn directly from the drain and not the collection bag.
Diabetic patient	Preoperative HbA1c and blood glucose (51). Delay RIRS if blood glucose exceeds 400 mg/dL (51).
Artificial joints	If placed within 2 years, consider antibiotic prophylaxis for several days prior to RIRS (14)
Patients with other known risk factors for infectious complications following RIRS and complex patients with multiple comorbidities	Employ a multidisciplinary approach with input from the appropriate medical/surgical services on how to best manage the unique risk factors

## Intra-Operative Considerations

The current AUA guidelines recommend antibiotic prophylaxis for gram-negative rods and *Enterococci* species for patients undergoing upper urinary tract endoscopic procedures (14). Per the AUA, the perioperative antimicrobials of choice for RIRS are trimethoprim-sulfamethoxazole (TMP-SMX) or a 1st/2nd generation cephalosporin. Alternative antibiotic regiments are an aminoglycoside +/– ampicillin, aztreonam +/– ampicillin, or amoxicillin/clavulanate (14). The EAU guidelines are similar in their recommendation of TMP-SMX, an aminopenicillin plus a beta-lactamase inhibitor, or a 2nd/3rd generation cephalosporin (56). TMP-SMX should be avoided in the pregnant patient and aztreonam should be reserved for patients with renal insufficiency and penicillin allergies (14, 49). Parenteral antibiotic prophylaxis should be administered within I h of the procedure, or 2 h if vancomycin is used (14). Contrary to these recommendations, Deng et al. found there to be no difference in the rates of post-operative febrile UTI with or without antibiotic prophylaxis for patients with negative urine cultures undergoing ureteroscopic lithotripsy in their meta-analysis of 4,591 patients across 11 different studies (57). However, there was a significantly lower risk of post-operative pyuria and bacteriuria with patients receiving a single dose of antibiotics prior to the procedure, with no difference between oral or IV agents (57). Similarly to this later finding, Knopf et al., that found a single dose of levofloxacin reduce the risk of post-operative bacteriuria from 12.5 to 1.8% (58).

This controversy is not new, and while the AUA guidelines are in favor of antibiotic prophylaxis, the European Association of Urology does not take a hardline stance on their use for all patients (57). Antibiotic stewardship is critical as we have already begun to see the rise of common uropathogens such as *E.coli* with resistance patterns to fluoroquinolones and TMP-SMX (28). This is a growing body of evidence that favors the limiting the use of empiric antibiotic therapies both in an effort to limit the proliferation of resistance but also with potentially lowering rates of sepsis (27). Zisman et al. found in their retrospective study that a significant portion of patients with a positive urine culture prior to RIRS contained ciprofloxacin resistant pathogens (27). They aimed to tailor antibiotic prophylaxis with two agents based on their hospital's local resistance patterns, and found a decreased risk of septic events when compared with typical prophylaxis (27). Additionally, Schnabel et al. came to a similar conclusion during their literature review of antibiotic prophylaxis in urolithiasis, with moderate benefit to single dose prophylaxis in patients undergoing RIRS (59). This would suggest that perioperative antibiotics be given based on local sensitivities if they are known. Lastly, for fungal prophylaxis, the AUA best practice statement notes: “Single-dose antifungal prophylaxis is recommended for patients with asymptomatic funguria undergoing endoscopic, robotic, or open surgery on the urinary tract.” (14). Otherwise, there are no guidelines suggesting patients with specific risk factors received perioperative antifungal prophylaxis.

Maintaining low irrigation pressures during RIRS is key for reducing infectious complications. Pressure increases in the collecting system can impair renal filtration and even result in retrograde flow from the collecting system, known as “pyelovenous backflow” in which there is communication of urine and renal venous blood (60). Theoretically, this would allow for the communication of bacterial products and inflammatory mediators from the urinary tract to enter systemic circulation (45). This notion is supported by the fact that high intra-renal pressures experienced during RIRS has been found to be associated with post-operative fevers (60). Additionally, one *ex-vivo* study of simulated ureteroscopy found a link between high intra-renal pressures and histologic changes as well as fluid extravasation in a porcine model (60). Normal intrapelvic pressure are approximately 5 mmHg, and the threshold for pyelovenous reflux is approximately 35 mmHg (61). Intraoperatively, pressures can reach up to 328 mmHg during forced irrigation, almost 10 times the reflux threshold (61).

A commonly used method for maintaining low intrapelvic pressure and reducing the risk of infectious complications is the use of a ureteral access sheath (UAS) (61). A UAS is an instrument originally conceived as a “guide tube” used during ureteroscopy for repeated entry to the ureter and renal collecting system while maintaining lower irrigations pressures (61). A UAS facilitates low irrigation pressures because it creates a channel from the collection system to outside of the body. This channel allows for irrigation outflow and equilibration with atmospheric pressure. Notably, these effects are most pronounced when a large diameter sheath is used and instruments are not obstructing the lumen (61). UAS have been shown to reduce intrapelvic pressure by up to 75% and large diameter sheaths can maintain pressures below the reflux threshold for the duration of the procedure (61). When using a UAS during RIRS, intrapelvic pressure is inversely related to UAS and directly related to ureteroscope size (61).

UAS may also reduce infection risk by minimizing operative time. Kim et al. found operative time to be an independent risk factor for the development of a febrile UTI following RIRS on multivariate analysis (62). This could be related to several factors, namely stone burden, irrigation pressure and irrigation volume (45, 62). Reasonably, increased operating time require the use of a larger amount of irrigation volume when compared to shorter procedures. A higher stone burden would also necessitate more operative time and provide more nidi for infection (45, 62). The continued introduction of foreign fluid into the genitourinary system, coupled with the repeated exposure of the internal matrix of the stone would provide an avenue for infection (45). If this were done under high pressure, the likelihood of the patient experiencing the movement of infectious material into other systems via pyelovenous reflux also would increase (45). One method that has been shown to reduce operative time is the use of an UAS secondary to the reduce time for repeated entry into the collecting system (61). However, the use of a UAS is not without its own risks, as their use can cause independent damage to the ureters ranging from superficial lesions of the mucosa to circumferential perforations (61).

Another method for maintaining low irrigation pressures is the use of gravity irrigation. A retrospective study by Farag et al. suggested that the use of fixed pressurized bag gravity irrigation was associated with less infectious complications when compared to the use of hand-syringe irrigation during RIRS (63). Gravity irrigation is “natural irrigation based on the height from the tip of the ureteroscope to the surface of saline” (64). With gravity irrigation, the saline flow rate depends on the height that the irrigation bag is hung, the height of the operating room table, and the size of the instrument that occupies the working channel (64). Smaller working channel diameters and shorter heights tend to lead to lower irrigation flow rates (64). One issue with the use of gravity irrigation is that a constant flowrate cannot be maintained as the pressure decreases as the bag empties and collecting system fills.

There are a variety of methods beings trialed allow for adequate flow rate while maintaining low collecting system pressures. These include automatic pumps, hand controlled syringes, and foot pedal controlled devices that seek to provide at constant flow rate at lower pressures (64). Inoue et al. sought to explore this further, and compared two novel automatic irrigation pumps to gravity irrigation in terms of flow rates at similar pressures (64). They found that with and without instruments gravity irrigation was had consistently lower flow rates compared to one of the two automatic pumps in this *ex vivo* study (64). Hendlin et al. found in their *ex-vivo* study that gravity irrigation exerted less force than both hand and foot controlled pump devices (65). Ultimately, when selecting irrigation methodology, one should aim to use the minimum pressure that provides adequate visualization.

Intraoperative stone cultures are another tool used in the management of infectious complications. Retrieval of a stone for culture during RIRS under sterile conditions can provide vital information for future infectious complications (7). Bacteria can be situated within the matrix of the stone and therefore not be sample on preoperative urine cultures or be targeted by antibiotic prophylaxis (6, 7). Evidence to suggests that positive stone cultures are important predictors of infectious complications (7). If a patient develops post-operative infectious complications, stone culture results may help guide targeted antimicrobial therapy. For this reason we recommend sending stone cultures intraoperatively when there is suspicion that the stone may be an infection stone or infected stone. One should be aware that there is often discordance between preoperative cultures and stone cultures (66). Korets et al. aimed to determine the concordance between preoperative bladder cultures and intraoperative stone and renal pelvis cultures as well as infectious complications (66). This prospective study found that in patients with a positive preoperative bladder culture and a positive renal pelvis culture, concordance was 64.3% (66). Stone cultures were concordant with preoperative bladder cultures in 70.6% of patients with a positive result for both (66). In patients with a positive stone culture and renal pelvic culture there was 75% concordance (66).

Forced diuresis is another method that can be employed and may reduce the risk of infectious complications following RIRS (7). The main concept is that administering a diuretic agent such as furosemide intravenously while in the operating room may help to prevent pyelovenous reflux by increasing urine production and improving outflow during the procedure, though the evidence supporting this practice is relatively weak (7). As a final note on intraoperative management, when performing RIRS, it is important to maintain open lines of communication with the anesthesiology team. Often times anesthesiologists will be the first to see physiologic signs of impending sepsis which may require prompt termination of the procedure. This applied to all types of surgery, not just RIRS, as a team-based approach has been shown to improve outcomes for all surgical procedures (67). If there is intraoperative suspicion that an infectious complication may be developing or that the patient is at high risk, placement of a foley catheter and ureteral stent should be strongly considered for maximum urinary tract decompression. As a final note, when performing RIRS for nephrolithiasis, two common strategies for stone destruction are stone dusting and stone fragmenting. Currently, there are no high-quality prospective randomized studies comparing stone dusting to stone fragmenting in regards to infectious complications (most such comparative studies evaluate stone free rate as a primary outcome), and this represents an important avenue for future research. Intra-operative recommendations are summarized in Table 2.

**Table 2:** Summary of intra-operative considerations.

**Intervention**	**Mitigation strategy**
Perioperative antibiotic therapy	**AUA Guidelines** (14): Within 1 h of procedure, •TMP-SMX or a 1st/2nd generation cephalosporin. Alternatives: •aminoglycoside +/– ampicillin or •aztreonam +/– ampicillin or •amoxicillin/clavulanate. **EAU Guidelines** (56): •TMP •TMP-SMX •2nd or 3rd generation cephalosporin •Aminopenicillin + beta-lactamase inhibitor
Maintaining low intra-renal pressures to minimize pyelovenous backflow	•Ureteral access sheath (UAS) (61). •Gravity irrigation (63–65). •Low irrigation pressures when employing hand/foot-controlled systems (64)
Other intraoperative techniques	•Obtaining stone cultures to guide post-operative antibiotics (7). •Forced diuresis (7)

## Post-Operative Considerations

Even with optimal preoperative and intraoperative strategies, the development of infectious complications after RIRS in some patients is inevitable, and early recognition and treatment is crucial to minimize morbidity (7). Patients should be monitored in the recovery room and for patients with known risk factors for infectious complications, a prolonged recovery room stay may be warranted with possible admission for observation to monitor for postoperative sepsis (11). Sepsis is defined by the Third International Consensus Definition for Sepsis and Septic Shock (Sepsis-3) as “life-threatening organ dysfunction resulting from dysregulated host responses to infection” (68). Urosepsis is sepsis originating from the urinary tract (69). Clinical criteria for the diagnosis of sepsis is quantified by the sequential organ failure assessment (SOFA) scoring system (70). This system assigns a score from 0–4 to each of the six major organ systems, including respiratory, coagulation, hepatic, cardiovascular, central nervous and renal systems with a higher score indicating worse function (70). Sepsis is identified in patients with an acute change in SOFA score of 2 or greater in the presence of infection, typically in an intensive care setting (70). Outside of an ICU setting, the quick SOFA (qSOFA) is used for risk stratification for patients at risk for sepsis (70). This tool is comprised of three criteria: alteration in mental status (Glasgow coma scale), systolic blood pressure of <100 mmHg, and a respiratory rate of ≥22 breaths per minute (70). Patients are considered high risk if two or more criteria are met (70). In the context of stone disease, an inflammatory reaction can develop from the release of endotoxin secondary to stone fragmentation or from the release of bacteria. Bacteria and their surface molecules act as pathogen associated molecular patterns (PAMPs) that bind receptors on the surfaces of the innate immune system (69, 71). This stimulates a local immune reaction as well as the induction of the transcription of various inflammatory mediators (71). In the genitourinary system and elsewhere, this can lead to an initial overwhelming cascade of inflammation as more immune cells are recruited, local tissues are damages, and mediators such as nitrous oxide cause local edema (71).

If impending urosepsis is suspected, urine and blood cultures should be obtained, and antibiotics should be promptly initiated. Culture directed treatment is ideal, but antibiotics should not be delayed for culture results. Delayed antibiotic treatment is associated with increased mortality in patients with severe sepsis, so empiric therapy with broad-spectrum antibiotics should be started as soon as possible (66). Though most uropathogens are gram negative, gram positive bacteria are becoming an increasingly important source of urologic infections (29). Empiric antibiotics should cover both gram negative and gram-positive bacteria and options include various combinations of the following: ampicillin, gentamicin, piperacillin/tazobactam, carbapenems, cefepime, and vancomycin. However, in selecting antibiotics, special consideration must be given to extended spectrum beta-lactamase (ESBL) producing bacteria. One randomized clinical trial by Harris et al. found a higher 30-day mortality rate for ESBL bacteremic patients treated with piperacillin-tazobactam compared to patients treated with meropenem (12.3 vs 3.7%) (72). Options for treating ESBL include carbapenems alone or in combination with Fosfomycin or tigecycline (69).

Another important etiology of post-operative sepsis is a fungal infection. The criteria for initiating fungal treatment is vague, and is generally to initiate therapy in critically ill patients with known risk factors and no other cause of fever (73). Using an arbitrary cut off of greater than a 10% risk of infection to start antifungal therapy has been suggested in the literature (73). However, as a general rule, given the morbidity and mortality associated with fungal sepsis initiation of antifungal therapy should be strongly considered upon early clinical suspicion (73). Echinocandins may be considered for empiric therapy because of azole resistant organisms in patients with recent azole exposure or suspected *Candida glabrata* infection (73). Fluconazole may be considered in non-critically ill patients (73). Ultimately when a patient is suspecting of having post-operative urosepsis from RIRS, early consultation by infectious disease specialist should be obtained to determine the optimal empiric regiment based on local resistance patterns. Switching antibiotic therapy from empiric to therapy based sensitivities should be done as soon as the information becomes available (22). In addition to prompt initiation of antimicrobial therapy and infectious disease consultation, the diagnosis of urosepsis following RIRS should prompt an early escalation of care to an ICU setting (74). Patients should receive proper hemodynamic and respiratory support if their clinical condition necessitates it (69). Elimination of any nidus for infection should be done, if possible (69). Additionally, patients who do not have a foley catheter in place should have to catheter placed for maximum urinary tract decompression.

Urosepsis secondary to KSD carries a high risk of morbidity and mortality (12, 22, 69, 71) and accordingly routine use of postoperative antibiotics to minimize infectious complications has been an active area of discussion. The current guidelines state that there is no evidence to continue antibiotic therapy after 24 h in the absence of other factors (14). However, some centers continue antibiotic therapy anywhere from 3 to 5 days postoperatively even in patients with negative cultures (74). Given the rise in MDR bacteria, the use of postoperative antibiotics in patients without risk factors or evidence of postoperative infection should be limited. Post-operative recommendations are summarized in Table 3.

**Table 3:** Summary of post-operative considerations.

**Risk factor/intervention**	**Mitigation strategy**
Urosepsis (Bacterial)	Early recognition with qSOFA and SOFA scores (68, 70)
	Urine and Blood cultures
	Empiric therapy covering gram positive and gram negative pathogens (69) **One or more of the following:** ampicillin, gentamicin, piperacillin/tazobactam*, carbapenems, cefepime, and vancomycin *Poor efficacy for ESBL bacteremia (72)
	Obtain prompt infectious disease consult
	Early escalation of care to ICU
Sepsis (Fungal)	Suspect in critically ill patients with known risk factors and no other cause of symptoms (73)
	Start empiric therapy when there is any clinical suspicion of fungal sepsis as the associated morbidity and mortality is high (73)
	Empiric therapy with echinocandins in anticipation of azole resistance (73)
	Obtain prompt infectious disease consult
	Early escalation of care to ICU

## Conclusion

KSD remains a common medical ailment effectively treated by urologists with RIRS. While adverse events are rare, infectious complications can produce serious consequences. It is vital for clinicians to understand which patients are at risk for infectious complications and the steps that can be taken to minimize such complications. Furthermore, understanding and recognizing the warning signs of a serious infection postoperatively coupled with knowledge of current guidelines and the most effective treatments are critical to addressing these complications when they arise.

## Author Contributions

JK: conception and design, analysis and interpretation of data, drafting of manuscript, and supervision. JH: acquisition of data, analysis and interpretation of data, and drafting of manuscript. AS: conception and design, analysis and interpretation of data, and critical revision of manuscript for important intellectual content. WA and MG: conception and design, analysis and interpretation of data, critical revision of manuscript for important intellectual content, and supervision. All authors contributed to the article and approved the submittedversion.

## Conflict of Interest

MG is compensated for educational training for Cook Urological Inc., Boston Scientific Inc., Olympus Inc., Lumenis Inc., and Retrophin Inc. The remaining authors declare that the research was conducted in the absence of any commercial or financial relationships that could be construed as a potential conflict of interest.

## Publisher's Note

All claims expressed in this article are solely those of the authors and do not necessarily represent those of their affiliated organizations, or those of the publisher, the editors and the reviewers. Any product that may be evaluated in this article, or claim that may be made by its manufacturer, is not guaranteed or endorsed by the publisher.
